# Progression of Precancerous Cervical Lesion Predicted by p16 Protein Immunohistochemistry in Rajavithi Hospital

**DOI:** 10.31557/APJCP.2019.20.6.1809

**Published:** 2019

**Authors:** Tipnaree Charoonwatana, Sathone Boonlikit, Marut Yanaranop

**Affiliations:** *Department of Obstetrics and Gynecology, Rajavithi Hospital, Bangkok, Thailand. *

**Keywords:** p16 protein immunohistochemistry, cervical intraepithelial neoplasia, precancerous cervical lesion

## Abstract

**Objective:**

To assess the association of p16 immunohistochemical (IHC) staining in cervical squamous intraepithelial lesions (SIL) and progression of cervical intraepithelial neoplasia (CIN) 1 to CIN2+ or recurrence of CIN2+.

**Material and Methods:**

A retrospective cohort study of women with newly diagnosed SIL from colposcopy-directed biopsy at Rajavithi Hospital, 2013-2017. Pathologic specimens were reviewed and submitted to p16-IHC staining. Adjusted hazard ratios (HR) of disease-free interval (DFI) and 95% confidence intervals (CI) were carried out using the Cox proportional hazard regression model.

**Results:**

A total of 187 women was recruited, 91 cases of positive p16-IHC staining and 96 cases of negative staining. With the median follow-up time of 22 months, women with positive p16-IHC had significantly lower 1-year DFI than those with negative p16-IHC (86.8% vs. 96.6%, p = 0.006). Women with CIN 1 had 22.6% of positive p16-IHC, while those with CIN2-3 had 86.7%. From multivariate analysis, the positive p16-IHC and age > 35 years were the significant prognostic factors of progression/recurrent CIN2+ (adjusted HR 5.33, 95%CI 1.77-16.01, p = 0.003; and adjusted HR 5.80, 95%CI 1.34-25.08, p = 0.019, respectively). From subgroup analysis, the positive p16-IHC was the significant prognostic factor in women with initial CIN1 (HR 5.29, 95%CI 1.18-23.76, p = 0.030), but was not associated with prognosis in women with initial CIN 2-3 (HR 2.13, 95%CI 0.28-16.38, p = 0.468).

**Conclusion:**

Overexpression of p16 protein has the prognostic significance of SIL. Using p16-IHC may help stratify patients as low-risk and high-risk groups to progression/recurrence CIN2+.

## Introduction

Cervical cancer is the fourth most frequent cancer in women worldwide(Bruni et al., 2017) which remains a leading cause of cancer-related death for women in developing countries. As known, it is a well-controlled disease in industrialized countries because of Papanicolaou (PAP) test and effective screening program implementation. (Sherris et al., 2001; Catarino et al., 2015; Torre et al., 2017) Disease prevalence is decreasing in those population. Further attention should be paid in specific screening algorithm to enhance the efficacy of screening protocols. It is well known that cervical cancer is preceded by high grade intraepithelial lesion (HSIL) which follows persistent human papillomavirus (HPV) infection. 

In fact, active transcription of HPV oncogene can be directly detected by E6/E7 viral mRNA transcripts and indirectly detected by accumulation of the host protein p16 in the cell. It has been widely reported that p16 expression is affected by the high risk HPV E7 protein and its up-regulation increasing severity of cervical lesions. (Giarrè et al., 2001; Li et al., 2011) Protein p16, a tumor suppressor from the Ink4 family, encoded by CDKN2A gene (9p21.3) prevents progression into S phase of cell cycle by inhibits cyclin D dependent protein kinases (CDK4 and CDK6) therefore maintaining retinoblastoma protein (pRb) in its hypophosphorylated state which prevents its dissociation from E2F transcription factor. Integration of viral E7 oncoprotein integrate into host genome leads to inactivation of pRb and overexpression of p16, therefore p16 protein immunohistochemistry (p16 IHC) is surrogate marker of high risk HPV infection. (Sano et al., 1998; Klaes et al., 2001; Negri et al., 2004; Queriroz et al., 2006; Iaconis et al., 2007)

The main objective of the present study was to assess the association of the overexpression of p16 IHC and progression of cervical intraepithelial neoplasia (CIN) 1 to CIN2 or worse (CIN2+) or recurrence of CIN2+ after treatment during follow up in women who had a colposcopy-directed biopsy (CDB).

## Materials and Methods

A retrospective cohort study, the women who newly diagnosed SIL from CDB at the Department of Obstetrics and Gynecology of Rajavithi Hospital, Bangkok from January 2013 to June 2017, all the patients showing histologically confirmed precancerous cervical lesion at an initial evaluation and followed up at least 1 year from the date of histologic diagnosis by CBD to the last visit were included in this study. 

*Study design and Population*

Patients whom diagnosed precancerous cervical lesion from CBD including HPV CIN1 CIN2 CIN3 were recruited, H and E slide were recut and reviewed with blinding previous diagnosis. Then p16 IHC were stained and interpreted by same pathologist in different time. Positive staining was defined as “block” staining: strong abnormal nuclear and cytoplasmic expression in a continuous segment of cells (at least 10 - 20 cells); in squamous epithelium, block positivity needs to involve basal and parabasal layers. Cytoplasmic only staining, diffuse blush / weak intensity staining / other focal / patchy patterns should be considered negative as. (Darragh et al., 2012; Kurman et al., 2014) The exclusion criteria were previous history of SIL, cervical cancer and absent paraffin block. Patients who underwent see & treat strategy were not analyzed.

*Follow-up and Outcome*

All patients underwent standard treatment during followed up, when abnormal cytology screening (ASCUS or worse) was presented, cervical tissue was confirmed by CDB. After diagnosis of precancerous cervical lesion, patients with low-grade lesions (HPV or CIN1) were observed every 6 months by conventional pap test or Liquid-based cytology at each follow-up while patients with high-grade lesion had intervention as standard protocol either Loop Electrosurgical Excision Procedure (LEEP) or local ablation then patients were followed up with cytology every 6 months. If there was any abnormality, CDB was done to confirm histologic diagnosis but in case of normal colposcopy, CBD was not performed. The outcome was measured by disease free interval(DFI) which was the time from diagnosis to progression to CIN2+ in case HPV/CIN1 or the time from diagnosis to recurrence to CIN2+ in case CIN2-3 after treatment (Health, 2014).

*Statistical Analysis*

The study sample size was estimated based on the formula for two independent proportion formula of **Bernard (2000)???** with 1:1 ratio, a proportion in group 1 of 0.250 and group 2 of 0.029 from study of Razmpoosh et al., (2014) were incorporated into the calculation. All data was analyzed using STATA program fifteenth version, the descriptive analysis were described by mean, SD, median, range for numerical data and using proportion, percentage for categorical data. The inferential analysis was compared by Student t-test, Chi-square test, Fisher exact test. The 95% confidence intervals (CI) were carried out using the Cox proportional hazard regression model. A value of p < 0.05 was considered statistically significant.

## Results

The 263 women who newly diagnosed SIL were recruited, then 76 women who absented paraffin block were excluded. Finally, 187 women were analyzed by p16 IHC staining. Ninety-one women were positive for p16 IHC staining and 96 women were negative for p16 IHC staining. 

The baseline characteristics including age parity smoking and immunocompromised status were balance but initial histology and treatment were statistically significant difference as summarized in table1. The mean age of participants was 40.82 and 42.31 years in positive p16 and negative p16 staining respectively, most of them were multi-parity no smoking and no underlying immunocompromised status. Mostly, LSIL were negative p16 about 77.7% and progressed to CIN2+ only 3.6% while the progression of positive p16 LSIL was 16.7% In contrast, HSIL found 84.8% of HSIL had positive p16 and recurrence were 17.9% as shown in Table 2.

**Figure 1:**
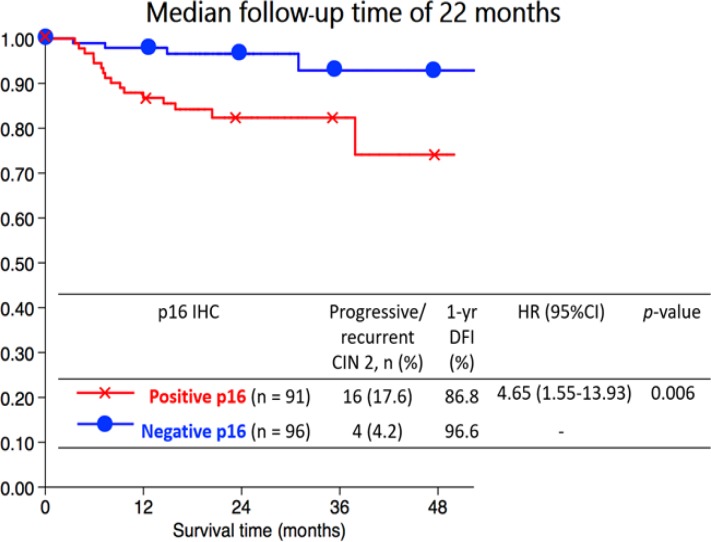
The Kaplan Meier Curve for 1 Year DFI of p16 IHC and Progressive/recurrent CIN2+

The Kaplan Meier curve for 1-year DFI showed that women who had positive p16 had significantly lower than negative p16 (86.8% vs. 96.6%; p = 0.003) with the median follow up time of 22 months as Figure 1. The univariate analysis of variable associated progression or recurrence to CIN2+ revealed that prognostic factors were age, initial histology, and p16 staining. After performing the multivariate analysis, it showed the p16 IHC and age > 35 years were significant prognostic factors for poor outcomes after adjust other factors (Table 3). 

**Figure 2:**
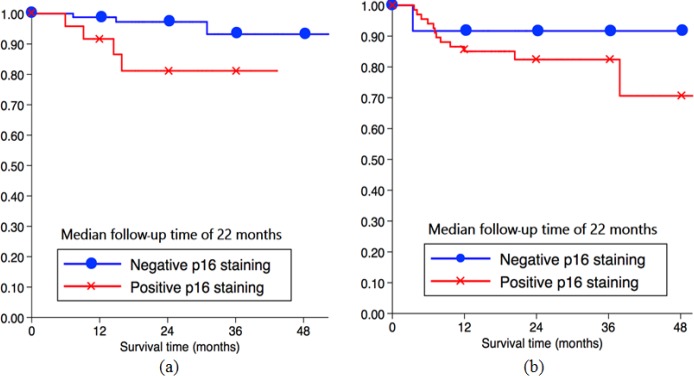
The Kaplan Meier Curve for 1 Year DFI of p16 IHC and Progressive CIN2+ in LSIL (a) and HSIL (b)

The Subgroup analysis of Prognostic Factors Related to Progressive CIN2+, positive p16 was important prognostic factor with significant difference in women diagnosed with low-grade lesion (Table 4). 1-year DFI in those with positive p16 IHC (91.7%) was lower than those with negtive p16 (98.8%) with significant difference, which was confirmed by the Kaplan Meier curve for 1-year DFI of p16 IHC and progressive CIN2+ in Figure 2a. However, in women diagnosed with high-grade lesion, positive p16 was not significantly associated recurrence to CIN2+ after treatment (Table 5) which shown in the Kaplan Meier curve of DFI in Figure 2b.

**Table 1:** The Baseline Characteristics

Characteristics	Positive p16		Negative p16		p-value
	n = 91 (%)		n = 96 (%)		
Age (yrs.), mean (SD)†	40.82	(12.5)	42.31	(11.1)	0.39
Parity ‡					
Nulliparity	17	(19.5)	15	(16)	0.563
Multiparity	74	(80.5)	79	(84)	
Immunocompromised status ‡	7	(7.7)	10	(10.4)	0.517
Smoking §	1	(1.1)	2	(2.1)	0.999
Histology ‡					
LSIL	24	(22.6)	84	(77.4)	< 0.001*
HSIL	67	(86.7)	12	(13.3)	
Treatment ‡					
None	33	(36.3)	72	(75)	< 0.001*
LEEP	55	(60.4)	24	(25)	
Hysterectomy	3	(3.3)	0	0	

HSIL, high-grade squamous intraepithelial lesion; LEEP, loop electrosurgical excision procedure; LSIL, low-grade squamous intraepithelial lesion; p16, p16 protein-immunohistochemistry; SD, standard deviation; *, Significance at p-value less than 0.05; †Student t-test, ‡, Chi-square test; §, Fisher-exact test.

**Table 2:** Progression of Precancerous Cervical Lesion Associated with p16 IHC Staining

		Total (%)	Progressive/recurrent CIN2+ (%)
LSIL	Negative p16 IHC	84 (77.7)	3 (3.6)
(N = 108)	Positive p16 IHC	24 (22.2)	4 (16.7)
HSIL	Negative p16 IHC	12 (15.2)	1 (8.3)
(N = 79)	Positive p16 IHC	67 (84.8)	12 (17.9)

CIN2+, cervical intraepithelial neoplasia 2 or worse; p16 IHC, p16 protein-immunohistochemistry staining

**Figure 3:**
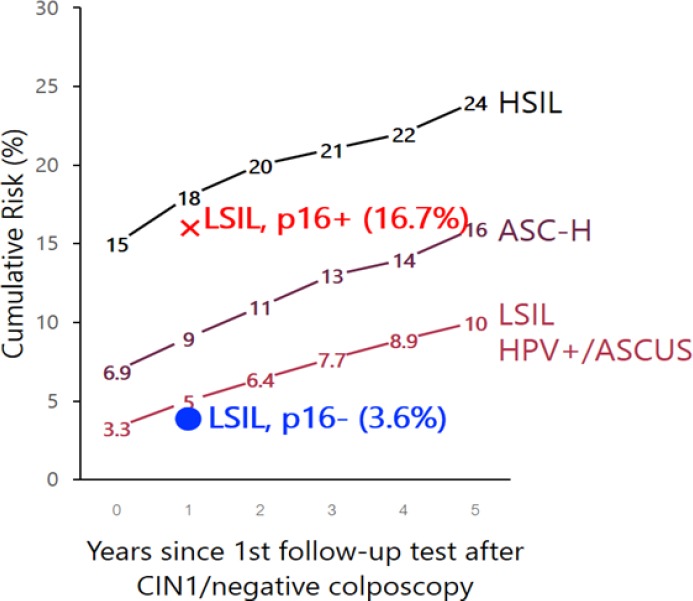
Benchmark of Cumulative Risk of CIN2+ Following CIN1/Negative Colposcopy Given Antecedent HSIL+, ASC-H, AGC, and LSIL/HPV+/ASC-US, among Women Aged 25 and Older (Katki et al, 2013). The progressive risk of LSIL with p16 staining positive (cross) and negative (dot) from this study

**Table 3:** Factor Associated with Progressive or Recurrent to CIN2 or Worse

Variables	N	Progressive/ recurrent CIN2+ (%)		Crude HR	95% CI	P-value	Adjusted HR	95% CI	P-value
Age									
< 35 yrs.	62	2	(3.2)						
> 35 yrs.	125	18	(14.4)	4.86	(1.13-20.97)	0.034*	5.8	(1.34-25.08)	0.019*
Parity									
Nulliparity	33	3	(9.1)						
Multiparity	154	17	(11)	1.41	(0.41-4.84)	0.588			
HIV infection									
No	170	20	(11.8)						
Yes	17	0	0						
Smoking									
No	184	20	(10.9)						
Yes	3	0	0						
Initial histology									
LSIL	108	7	(6.5)						
HSIL	79	13	(16.5)	2.8	(1.12-7.03)	0.028*			
p16 IHC staining									
Negative	96	4	(4.2)						
Positive	91	16	(17.6)	4.65	(1.55-13.93)	0.006*	5.33	(1.77-16.01)	0.003*
Treatment									
Observation	105	10	(9.5)						
LEEP	79	9	(11.4)	1.27	(0.52-3.13)	0.6			
Hysterectomy	3	1	(33.3)	4.42	(0.56-34.73)	0.158			

CIN2+, cervical intraepithelial neoplasia 2 or worse; HSIL, high-grade squamous intraepithelial lesion; LEEP, loop electrosurgical excision procedure; LSIL, low-grade squamous intraepithelial lesion; p16 IHC, p16 protein-immunohistochemistry; * Significance at p-value less than 0.05; †, Student t-test; ‡, Chi-square test, §Fisher-exact test

**Table 4:** Factor Associated with Progressive to CIN2 or Worse in LSIL

Variables	N	Progressive CIN2+ (%)		Crude HR	95% CI	P-value
Age						
< 35 yrs.	32	2	(6.25)			
> 35 yrs.	72	5	(6.94)	1.4	(0.27-7.26)	0.684
Parity						
Nulliparity	21	1	(4.76)			
Multiparity	87	6	(6.9)	1.54	(0.18-12.80)	0.691
HIV infection						
No	97	7	(7.22)			
Yes	11	0				
Smoking						
No	107	7	(6.54)			
Yes	1	0				
p16 IHC staining						
Negative	84	3	(3.57)			
Positive	24	4	(16.67)	5.29	(1.18-23.76)	0.030*
Treatment						
Observation	93	7	(7.53)			
LEEP	15	0				

CIN2+, cervical intraepithelial neoplasia 2 or worse; LEEP, loop electrosurgical excision procedure; p16 IHC, p16 protein-immunohistochemistry

*Significance at p-value less than 0.05

**Table 5:** Factor Associated with Recurrent to CIN2 or Worse in HSIL

Variables	N	Recurrent CIN2+ (%)		Crude HR	95% CI	P-value
Age						
< 50 yrs.	58	9	(15.51)			
> 50 yrs.	21	4	(19.05)	1.32	(0.41-4.29)	0.644
Parity						
Nulliparity	12	2	(16.67)			
Multiparity	67	11	(16.42)	1.29	(0.27-6.09)	0.743
HIV infection						
No	73	13	(17.81)			
Yes	6	0				
Smoking						
No	77	13	(16.88)			
Yes	2	0				
p16 IHC staining						
Negative	12	1	(8.33)			
Positive	67	12	(17.91)	2.13	(0.28-16.38)	0.468
Treatment						
Observation	12	3	(25)			
LEEP	64	9	(14.06)	0.64	(0.17-2.41)	0.511
Hysterectomy	3	1	(33.33)	1.84	(0.19-18.20)	0.601

CIN2+, cervical intraepithelial neoplasia 2 or worse; LEEP, loop electrosurgical excision procedure; p16 IHC, p16 protein-immunohistochemistry

*Significance at p-value less than 0.05

**Table 6:** Comparation of Previous Studies

Author (yr)	N	Baseline	p16 IHC (%)	F/U time (mo.)	Progress CIN2+	Results
Razmpoosh (2014)(15)	64	CIN1	27.4	> 6	25.00%	p = 0.002
Liao (2014)(16)	171	Normal	42.7	24	10.70%	RR 8.25 (1.02-66.62)
		CIN1				
Pacchiarotti (2014)(17)	124	Normal	50.8	24	10.30%	RR 5.20 (0.60-47.50)
		CIN1				
Sagasta (2016)(18)	416	HPV	11.3	28	22.40%	HR 1.6 (0.9–2.6)
		CIN1				
This study	108	HPV	22.2	22	16.70%	HR 5.29 (1.18-23.76)
		CIN1				

## Discussion

In the present study, SIL with positive p16 staining was likely progressed or recurred to CIN2+ with significant difference. Therefore, it is possible that p16 overexpression might be used as a predictor among the patients who diagnosed with SIL. Low-grade lesion with positive p16 staining was likely to progressed about 5.29 times compared to those with negative p16 staining. From previous studies, approximately 10-25% of CIN1 with positive p16 staining progressed to CIN2+ (Table 6) which were similar to our study. However, the results from those studies were inconclusive. The statistical difference was obvious in 2 studies (Liao et al., 2014; Razmpoosh et al., 2014) whereas not statistically different in another 2 studies (Pacchiarotti et al., 2014; Sagasta et al., 2016). In the present study, high-grade lesion with positive p16 staining had recurrence to CIN2+ about 2.13 times than those with negative p16 staining but it was not significantly different. It is well known that status of LEEP’s margin predicts the outcome of treatment, women who have positive excisional margin trend to recur more than those who have negative margin. In the present study, the proportion of women who have positive margin was not identified therefore we do not know exactly how many patients at risk for recurrence in our population. If p16 test is performed in particular group such as women with positive margin, it may be useful and further study in this area is needed.

Base on the concept of equal management of equal risk theory (Katki et al., 2013) in Figure 5, progressive risk of LSIL with negative p16 staining (dot) from the present study is lower than benchmark of Cumulative risk of CIN2+ at 1 year after CIN1/ negative colposcopy by antecedent LSIL / positive HPV DNA ASCUS. These patient might be reassured for follow up without treatment by cotesting at 1 year later followed by ASCCP guideline (Massad et al., 2013). While the progressive risk of LSIL with positive p16 staining (cross) is higher than benchmark (Katki et al., 2013) therefore these patient may benefit more from excision procedure but further studies in larger population is required to confirm these findings.

The important strength of the present study is all slides were histologically reviewed for decrease selective bias and the p16 IHC staining and H and E slides were interpreted by the same pathologist in different time and blinding of result. The present study has some limitation. First, the retrospective study leads to incomplete data collected and information bias. Second, a significant loss of paraffin blocks might render the data not representing the actual population.

In conclusion, the overexpression of p16 protein was the significant prognostic factor of SIL. By using the p16 IHC may help stratify patients as low-risk and high-risk groups to predict progression or recurrence CIN2+. 

## Funding Statement

The present study was supported by the research fund from Rajavithi Hospital, Department of medical services.

## Conflict of interest

The authors declare that they have no conflict of interest.
